# Stealthy Secret Key Generation

**DOI:** 10.3390/e22060679

**Published:** 2020-06-18

**Authors:** Pin-Hsun Lin, Carsten R. Janda, Eduard A. Jorswieck, Rafael F. Schaefer

**Affiliations:** 1 Information Theory and Communication Systems Department, Technische Universität Braunschweig, 38106 Braunschweig, Germany; Janda@ifn.ing.tu-bs.de (C.R.J.); Jorswieck@ifn.ing.tu-bs.de (E.A.J.); 2 Information Theory and Applications Chair, Technische Universität Berlin, 10623 Berlin, Germany; rafael.schaefer@tu-berlin.de

**Keywords:** secret key generation, source model, stealthy communications, covert communications, channel resolvability, conceptual wiretap channel, stochastically degraded, stochastic orders

## Abstract

In order to make a warden, Willie, unaware of the existence of meaningful communications, there have been different schemes proposed including covert and stealth communications. When legitimate users have no channel advantage over Willie, the legitimate users may need additional secret keys to confuse Willie, if the stealth or covert communication is still possible. However, secret key generation (SKG) may raise Willie’s attention since it has a public discussion, which is observable by Willie. To prevent Willie’s attention, we consider the source model for SKG under a strong secrecy constraint, which has further to fulfill a stealth constraint. Our first contribution is that, if the stochastic dependence between the observations at Alice and Bob fulfills the strict more capable criterion with respect to the stochastic dependence between the observations at Alice and Willie or between Bob and Willie, then a positive stealthy secret key rate is identical to the one without the stealth constraint. Our second contribution is that, if the random variables observed at Alice, Bob, and Willie induced by the common random source form a Markov chain, then the key capacity of the source model SKG with the strong secrecy constraint and the stealth constraint is equal to the key capacity with the strong secrecy constraint, but without the stealth constraint. For the case of fast fading models, a sufficient condition for the existence of an equivalent model, which is degraded, is provided, based on stochastic orders. Furthermore, we present an example to illustrate our results.

## 1. Introduction

Consider the following motivating example. Two agents, Alice and Bob, want to establish a communication that does not raise the curiosity of a warden Willie, whose duty is to monitor if there is any suspicious activity and also decrypts the data. In order to realize a confidential transmission for such a scenario, we may adopt the following two steps. The first step is to make Willie unaware of the existence of the meaningful communication, which is embedded in the messages intended to be delivered to Bob. In contrast, in a meaningless communication, Bob does not care about the received signal, which is only used to confuse Willie. If Willie can successfully detect the existence of the meaningful transmission, then the second step is to use wiretap coding [1] to provide secrecy (or hidability [2]). There are two main concepts to attain the goal of the first step: (1) communications with a stealth constraint [2,3] and (2) communications with a covert constraint [2,4,5]. Both concepts make Willie unable to differentiate between the existence or nonexistence of the meaningful transmission, solely according to the probability distributions of his observations. More specifically, in the first concept, we transmit meaningful and meaningless signals non-overlapped in time. Note that the meaningful signal is the one Alice wants to communicate with Bob, while the meaningless signal is used to confuse Willie. If well designed, Willie cannot differentiate between those two signals, because the induced output distributions are close (the closeness can be defined in several different ways, e.g., by total variational distance, divergence, etc.) to each other. In the second concept, the meaningful signal can be superimposed on the meaningless one. Under the stealth constraint, we can have a positive capacity, while the covert transmission rate is zero, asymptotically. Even though the transmission rate of the second concept is in general zero, asymptotically, the second order rate is positive following the square root law [5,6].

For the aforementioned two concepts, if the channel between Alice and Bob (denoted by Bob’s channel in the following) is no better than the channel between Alice and Willie (denoted by Willie’s channel in the following), we need additional keys to conceal the meaningful signals, e.g., [4,5]. In particular, these additional keys are used to choose between codebooks to fool Willie. Our motivation is to design an achievable scheme for the source model secret key generation (SKG) for the above scenario, i.e., Bob has no channel advantage over Willie, while fulfilling both the security and stealth constraints, simultaneously. Note that the keys generated from the stealthy SKG can also be used to protect the data on top of concealing the behavior of transmission, e.g., encryption/one-time pad, etc. Note also that our design goal is violated if we directly apply common SKG schemes [7]. This is because common SKG schemes use public communications for several important operations including advantage distillation, information reconciliation, and privacy amplification ([7] Chapter 4.3).Without subtle modifications, these operations will raise Willie’s curiosity. To attain our objective, we focus on stealthy SKG, which is from its counterpart, stealth communications [3]. The main reason not to consider covertness but stealth for the SKG is that, under the assumption of a noiseless public discussion channel, there is no ambient noise to hide the discussion signal. Instead, covert SKG may be feasible if there is a noisy public channel. In addition, in general, the covert SKG suffers a sub-linear rate, e.g., [8], which is inherited from the covert communications. Recall that a channel-model SKG with a rate-unlimited public channel was considered in [8]. The authors applied the scheme from covert communication to the key transmission, while a stealth-like public discussion was used.

The main contributions of this work are summarized in the following:

- We investigate a source-model SKG under strong secrecy with an additional stealth constraint.
- We derive an achievable secret key (SK) rate under the stealth constraint, if I(X;Y)≥I(X;Z), where *X*, *Y*, and *Z* are the observations of the common randomness source at Alice, Bob, and Willie, respectively. Moreover, if (X,Y,Z) form a Markov chain X−Y−Z, then the SK capacity with the additional stealth constraint can be achieved without extra cost, compared to the SKG without the stealth constraint.
- We prove that a sufficient condition to achieve the stealthy SK capacity can be relaxed from the physically degraded channel to a stochastically degraded one.
- A sufficient condition for the existence of an equivalent degraded model is derived by the usual stochastic order [9], which is for the fast fading Gaussian Maurer’s (satellite) model [10].

Notation: Lower case bold letters denote deterministic vectors, and upper case normal/bold letters denote random variables/random vectors (or matrices), which will be defined when they are first mentioned. We denote the probability mass function (pmf) by *P*. The entropy of *X* is denoted as H(X). The mutual information between two random variables *X* and *Y* is denoted by I(X;Y). The divergence between distributions $P_{X}$ and $P_{Y}$ is denoted by $D(P_{X}||P_{Y})$. X∼F denotes that the random variable *X* follows the distribution *F*, while $\bar{F}≜1-F.$ The subscript *i* in $X_{i}$ denotes the *i*th symbol, and $X^{i}≜\left[X_{1},\;X_{2},\;\cdots ,\;X_{i}\right]$. X−Y−Z denotes the Markov chain. ⌈·⌉ denotes the ceiling operator. All logarithms are to base two. ${\left(a\right)}^{+}≜\max\left(a,0\right)$.

The rest of the paper is organized as follows. In Section 2, we introduce the preliminaries and the considered system model. In Section 3, we derive our main results. Finally, Section 4 concludes this paper.

## 2. Preliminaries and System Model

### 2.1. Preliminaries

We first introduce some necessary definitions and results to develop our work.

**Definition** **1.** *The strong secrecy and the stealth constraints are respectively defined as:*

$$\begin{array}{cc} D(P_{MZ^{n}}||P_{M}P_{Z^{n}}) & \leq \epsilon ,\\ D(P_{Z^{n}}||Q_{Z^{n}}) & \leq \epsilon , \end{array}$$

*for arbitrarily small ϵ>0, where M, $Z^{n}$, $P_{Z^{n}}$, and $Q_{Z^{n}}$ are transmitted messages, the observed signal at Willie, and the output distributions at Willie induced by meaningful and meaningless signals, respectively.*

The second constraint in the above definition can be explained by hypothesis testing as discussed in [3]. By this viewpoint, if the second constraint is fulfilled, the adversary’s best strategy is to blindly guess whether the current transmitted signal is meaningful or meaningless.

**Definition** **2.** *Denote a common random source as $\left(X,Y,Z,P_{XYZ}\right)$, where X,Y,andZ are the alphabets of the observations at Alice, Bob, and Willie. The random source is stochastically degraded, if the marginal distributions $P_{Y|X}$ and $P_{Z|X}$ are identical to those of another source of common randomness $\left(X,Y,Z,P_{X\tilde{Y}\tilde{Z}}\right)$ following the physical degradedness, i.e., $X-\tilde{Y}-\tilde{Z}$.*

**Corollary** **1.** *The same marginal property for one transmitter ([11] Theorem 13.9) Consider a discrete memoryless multiuser channel including one transmitter and two non-cooperative receivers with input and output alphabets X and Y×Z, respectively. The capacity region of such a channel depends only on the conditional marginal distributions $P_{Y|X}$ and $P_{Z|X}$ and not on the joint conditional distribution $P_{Y,Z|X}$, where X∈X and Y∈Y and Z∈Z are the transmit signal and the two receive signals, respectively.*

**Definition** **3.** *δ-robust typicality ([12] Appendix)*

*The sequence $x^{n}\in X^{n}$ is δ-robust typical for δ>0:*

(1) $$\begin{array}{c} T_{\delta }^{\left(n\right)}\left(P_{X}\right)=\left\{x^{n}\in X^{n}:\;\left|\frac{N(a|x^{n})}{n}-P_{X}\left(a\right)\right|\leq \delta P_{X}\left(a\right),\;\forall a\in X\right\}, \end{array}$$

*where $N(a|x^{n})$ is the number of occurrences of a in $x^{n}$.*

**Definition** **4.**  *([9] (1.A.3) For random variables A and B, $A\leq_{st}B$ if and only if ${\bar{F}}_{A}\left(a\right)\leq {\bar{F}}_{B}\left(a\right)$ for all a.*

Let $A{=}_{st}A^{\prime }$ denote that *A* and $A^{\prime }$ have the same distribution.

**Lemma** **1.** *Coupling [13]: $A\leq_{st}B$ if and only if there exists random variables $\hat{A}{=}_{st}A$ and $\hat{B}{=}_{st}B$ such that $\hat{A}\leq \hat{B}$ almost surely.*

### 2.2. System Model

**Figure 1:**
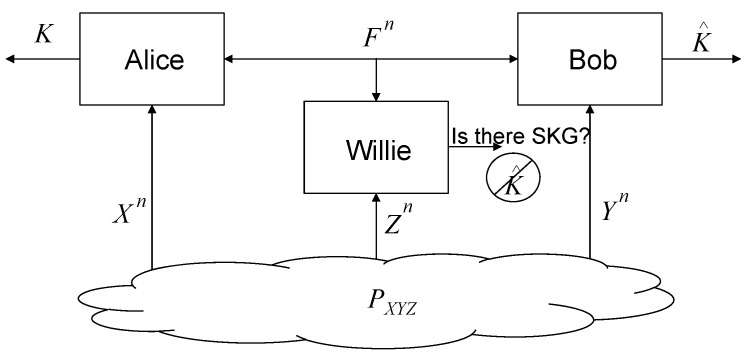
The system model of the considered stealthy secret key generation (SKG).

The considered system model is shown in Figure 1. We denote the *n*-time source observations at Alice, Bob, and Willie by $X^{n}$, $Y^{n}$, and $Z^{n}$, respectively, which follow the independent and identically distributed (i.i.d.) joint distribution $P_{X^{n}Y^{n}Z^{n}}=\;\prod_{i=1}^{n}P_{X_{i}Y_{i}Z_{i}}=\;\prod_{i=1}^{n}P_{XYZ}$ with alphabets X,Y,Z, respectively. The public discussion between Alice and Bob through a noiseless channel is denoted by a random vector $F^{n}\in F^{n}$. We consider the case without rate limitation on the public discussion channel. Willie can perfectly observe $F^{n}$. The joint distributions of the signals that Willie can observe when the SKG is meaningful and meaningless are denoted by $P_{F^{n}Z^{n}}$ and $Q_{F^{n}Z^{n}}$, respectively. Alice and Bob aim at sharing keys K∈K satisfying the constraints as follows:

(2) $$\begin{array}{cc} P_{e}≜Pr\left(K\neq \hat{K}\right) & \leq \epsilon , \end{array}$$

(3) $$\begin{array}{cc} \log|K|-H(K) & \leq \epsilon , \end{array}$$

(4) $$\begin{array}{cc} D(P_{KZ^{n}F^{n}}||P_{K}P_{Z^{n}F^{n}}) & \leq \epsilon , \end{array}$$

(5) $$\begin{array}{cc} D(P_{F^{n}Z^{n}}||Q_{F^{n}Z^{n}}) & \leq \epsilon \end{array}$$

for arbitrarily small ϵ>0, where (2) is the error probability having different keys at Bob from Alice, (3) is the keys’ uniformity constraint, while |K| is the number of keys and (4) is the constraint for the strong secret key, which is an adaptation from the stealth communication in Definition 1. In particular, *K* is dual to *M*, and $Z^{n}F^{n}$ is dual to $Z^{n}$. The stealth constraint is considered in (5), which is again an adaptation from Definition 1, i.e., here, $F^{n}Z^{n}$ is what Willie can observe, instead of solely $Z^{n}$ in stealth communications.

**Definition** **5.** *The rate of the keys generated fulfilling (2)–(5) is called the achievable stealthy strong SK rate.*

**Definition** **6.** *The maximum achievable stealthy strong SK rate is called the stealthy strong SK capacity.*

## 3. Main Results

We show two main result in this section: (1) the stealthy strong SK rate and a condition to attain the capacity; (2) a scheme to identify the fast fading Gaussian Maurer’s model as a degraded one, so that the stealth SK capacity can be determined explicitly.

### 3.1. Stealthy Strong Secret Key Rate and Capacity

Our main result is described by the following theorem followed by discussions.

**Theorem** **1.** *If (X,Y,Z) drawn from the common random source $\left(X,Y,Z,P_{XYZ}\right)$, then the stealthy strong SK capacity $C_{SK}$ of source model SKG with the stealth constraint can be bounded by:*

(6) $$\begin{array}{c} \max\left\{I\left(X;Y\right)-I\left(X;Z\right),\;I\left(Y;X\right)-I\left(Y;Z\right)\right\}\leq C_{SK}\leq \min\left\{I\left(X;Y|Z\right),\;I\left(X;Y\right)\right\}. \end{array}$$

*Furthermore, if (X,Y,Z) forms a Markov chain X−Y−Z, the stealthy strong SK capacity is:*

(7) $$\begin{array}{c} C_{SK}=I\left(X;Y\right)-I\left(X;Z\right). \end{array}$$

### 3.2. Sufficient Conditions for a Degraded Common Randomness

In this section, we derive a sufficient condition to obtain $C_{SK}=I\left(X;Y\right)-I\left(X;Z\right)$. In particular, we show that this sufficient condition, i.e., the common randomness forming a Markov chain X−Y−Z, can be relaxed to be stochastically degraded. After that, we show that this relaxed condition can be satisfied under a quite common setting by, e.g., the fast fading Gaussian Maurer’s (satellite) model [10]. In particular, a central random source $S_{0}$ emits signals passing through fast fading additive white Gaussian noise (AWGN) channels, which are observed as *X*, *Y*, and *Z* at Alice, Bob, and Willie, respectively.

**Theorem** **2.** *If a common random source $\left(X,Y,Z,P_{X\tilde{Y}\tilde{Z}}\right)$ is stochastically degraded such that $P_{\tilde{Y}|X}=P_{Y|X}$ and $P_{\tilde{Z}|X}=P_{Z|X}$, where X−Y−Z, then:*

(8) $$\begin{array}{c} C_{SK}=I\left(X;Y\right)-I\left(X;Z\right). \end{array}$$

The proof is delegated to Appendix C.

Example: Consider the fast fading Gaussian Maurer’s (satellite) model [10] as a special case of Theorem 2:

(9a) $$\begin{array}{cc} X & =A_{X}S_{0}+N_{X}, \end{array}$$

(9b) $$\begin{array}{cc} Y & =A_{Y}S_{0}+N_{Y}, \end{array}$$

(9c) $$\begin{array}{cc} Z & =A_{Z}S_{0}+N_{Z}, \end{array}$$

where $N_{X}$, $N_{Y}$, and $N_{Z}$ are independent AWGNs at Bob and Willie, respectively, while both are with zero mean and unit variance; $A_{X}$, $A_{Y}$, and $A_{Z}$ follow CDFs $F_{X}$, $F_{Y}$, and $F_{Z}$, respectively, and are the i.i.d. fast fading channel gains from the source $S_{0}$ to Alice and Willie, respectively. Note that intuitively, *X*, *Y*, and *Z* have no degradedness relation in general due to the random fading. This is because, by the definition of degradedness, the trichotomy order of all realizations between the two fading channels within a codeword length should be the same. We can invoke the same marginal property [14] to construct an equivalent channel, wherein by imposing the usual stochastic order constraint, we can identify those fading channels that can be re-ordered in the equivalent channel to keep the trichotomy order fixed.

If the random channels $A_{X}$ and $A_{Z}$ fulfill ${\bar{F}}_{A_{X}^{2}}\left(x\right)\geq {\bar{F}}_{A_{Z}^{2}}\left(x\right)$ for all *x*, where the subscripts denote the absolute square of the channel magnitudes, then from Lemma 1, we have equivalent (in the sense of having the same stealthy SK capacity) observations at Bob and Willie as $\hat{Y}={\hat{A}}_{X}S_{0}+N_{Y}$ and $\hat{Z}={\hat{A}}_{Z}S_{0}+N_{Z}$, respectively, where ${\hat{A}}_{X}^{2}\geq {\hat{A}}_{Z}^{2}$ almost surely. Therefore, it is clear that ${\bar{F}}_{A_{X}^{2}}\left(x\right)\geq {\bar{F}}_{A_{Z}^{2}}\left(x\right)$ is a relaxed sufficient condition to guarantee that *Z* is an equivalently stochastically degraded version of *Y*.

Assume that $A_{X}$ and $A_{Z}$ in Equations (9a)–(9c) are fast fading magnitudes following the Nakagami-*m* distribution with shape parameters $m_{x}$ and $m_{z}$ and spread parameters $w_{x}$ and $w_{z}$ [15], respectively. From Theorem 2, we know that *Z* is a degraded version of *X* if:

$$\begin{array}{c} \gamma \left(m_{x},\frac{m_{x}}{w_{x}}x\right)\Gamma \left(m_{z}\right)\geq \gamma \left(m_{z},\frac{m_{z}}{w_{z}}x\right)\Gamma \left(m_{x}\right),\;\forall x, \end{array}$$

where $\gamma \left(s,x\right)=\int_{0}^{x}t^{s-1}e^{-t}dt$ is the incomplete gamma function and $\Gamma \left(s\right)=\int_{0}^{\infty }t^{s-1}e^{-t}dt$ is the ordinary gamma function. An example satisfying the above inequality is $\left(m_{x},w_{x}\right)=\left(1,3\right)$ and $\left(m_{z},w_{z}\right)=\left(1,2\right)$.

## 4. Conclusions

In this work, we analyzed the performance of the secret key generation from a common random source, which satisfied the additional constraint that the generation of keys should not invoke the warden Willie’s attention. Our results showed that compared to the normal SKG, the additional stealth constraint could be fulfilled without extra cost. In particular, the stealthy SK capacity with strong secrecy constraint is I(X;Y)−I(X;Z), if the common random source satisfies I(X;Y)≥I(X;Z). To emphasize the practical relevance, a sufficient condition was derived to attain the degradedness by the usual stochastic order for the Gaussian Maurer’s (satellite) model for the source of common randomness under fast fading. As a final note, we can also use Slepian–Wolf coding with a proper use of the binning code book to derive the same result.

## Figures and Tables

**Figure A1:**
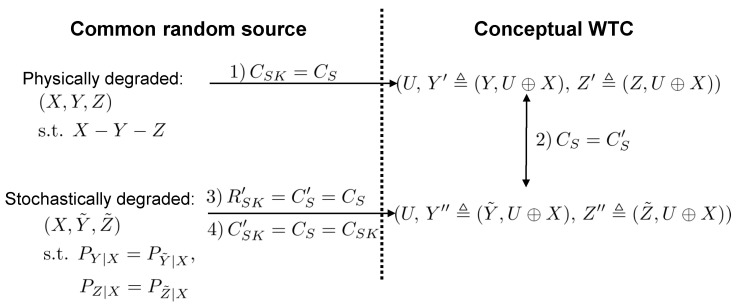
Key steps in the proof of Theorem 2. First is to show that the CWTCalso physically degraded if the source is physically degraded. Then, we show that the CWTC constructed from a stochastically degraded source corresponding to the physically degraded source is a stochastically degraded CWTC with the same marginal as that physically degraded CWTC. Finally, we show that the secrecy capacity of the second CWTC is indeed the secret key capacity of the stochastically degraded source. The key and secrecy capacities of the physically degraded source and the corresponding CWTC are denoted by $C_{SK}$ and $C_{S}$, respectively, while the key (rate) and secrecy capacities of the stochastic source and the corresponding CWTC are denoted by $C_{SK}^{\prime }\;\left(R_{SK}^{\prime }\right)$ and $C_{S}^{\prime }$, respectively.

## Appendix A. Proof of Theorem 1

Our main idea for deriving the lower bound of the SK capacity in the second scheme is by constructing a conceptual WTC (CWTC) as in [16]. An equivalent wiretap codebook $\left\{U^{n}\left(m,w\right)\right\}$ is constructed, where $m=1,\cdots ,L_{0}$, $w=1,\cdots ,L_{1}$, $L_{0}≜2^{nR}$ is the number of secure messages, and $L_{1}≜2^{nR_{1}}$ is the number of confusion messages; *m* and *w* are uniformly and independently selected; $U^{n}\left(m,w\right)\in X^{n},\;\forall \left(m,w\right)$. In addition, $\left(Z^{n},F^{n},U^{n}\right)$ are generated according to $P_{Z^{n}F^{n}U^{n}}=\prod_{i=1}^{n}P_{Z_{i}F_{i}U_{i}}=\prod_{i=1}^{n}P_{Z_{i}F_{i}|U_{i}}P_{U_{i}}$, where we consider the equivalent channel from Alice to Willie as:

(A1) $$\begin{array}{cc} P_{Z^{n}F^{n}|U^{n}} & =\prod_{i=1}^{n}P_{Z_{i}F_{i}|U_{i}}, \end{array}$$

where $\left(Z^{n},\;F^{n}\right)$ is the equivalent channel output at Willie. Similarly, $\left(Y^{n},\;F^{n}\right)$ is the equivalent channel output at Bob. We choose $U^{n}$ mutually independent of $X^{n},\;Y^{n}$, and $Z^{n}$.

In order to analyze the stealth, the respective distributions of the meaningful and meaningless signals at the equivalent channel output at Willie are:

(A3) $$P_{Z^{n}F^{n}}=\frac{1}{L_{0}L_{1}}\sum_{m=1}^{L_{0}}\sum_{w=1}^{L_{1}}P_{Z^{n},F^{n}|U^{n}}\left(z^{n},f^{n}|u^{n}\left(m,w\right)\right),$$

(A3) $$Q_{Z^{n}F^{n}}=\sum_{u^{n}}P_{Z^{n},F^{n}|U^{n}}\left(z^{n},f^{n}|u^{n}\right)P_{U^{n}}\left(u^{n}\right).$$

We first decompose the stealth secrecy constraint as follows:

(A4) $$\begin{array}{cc} \; & D(P_{KZ^{n}F^{n}}\left|\right|P_{K}P_{Z^{n}F^{n}})+D(P_{Z^{n}F^{n}}\left|\right|Q_{Z^{n}F^{n}})\\ = & \sum_{K,Z^{n},F^{n}}P_{KZ^{n}F^{n}}\left(\log\frac{P_{KZ^{n}F^{n}}}{P_{K}P_{Z^{n}F^{n}}}+\log\frac{P_{Z^{n}F^{n}}}{Q_{Z^{n}F^{n}}}\right)\\ = & \sum_{K,Z^{n},F^{n}}P_{KZ^{n}F^{n}}\left(\log\frac{P_{KZ^{n}F^{n}}}{P_{K}Q_{Z^{n}F^{n}}}\right)\\ ≜ & D(P_{KZ^{n}F^{n}}||P_{K}Q_{Z^{n}F^{n}}) \end{array}$$

(A5) $$\begin{array}{c} \overset{\left(a\right)}{=}D(P_{K}\left|\right|P_{K})+D(P_{Z^{n}F^{n}|K}\left|\right|Q_{Z^{n}F^{n}}\left|P_{K}\right)\\ =D(P_{Z^{n}F^{n}|K}||Q_{Z^{n}F^{n}}|P_{K}), \end{array}$$

where (a) follows the chain rule of divergence ([17] Th.2.2.2).

We then apply the channel resolvability analysis [18] to the CWTC, in order to find the rate constraint on the confusion messages, in order to guarantee the validity of the stealth secrecy constraint (A4).

From the analysis conducted in Appendix B, we know:

(A6) $$\begin{array}{c} \;\;E_{C}[D(P_{Z^{n}F^{n}|K}\left|\right|Q_{Z^{n}F^{n}}|P_{K})]\;\leq \;E_{Z^{n}F^{n}U^{n}}\left[\;\log\;\left(\;\frac{P_{Z^{n}F^{n}|U^{n}}}{L_{1}Q_{Z^{n}F^{n}}}\;+\;1\;\right)\;\right]. \end{array}$$

Recall that $L_{1}=2^{\left\lceil nR_{1}\right\rceil }$ is the number of confusion messages inside each bin, and we need to design it such that (A6) is vanishing. The main difference between our proof and that in [3] is that we introduce an additional channel output at both Bob and Willie by constructing a CWTC for the considered SKG model, which makes the results from [3] not able to be directly applied.

Now, we reexpress the ratio in the logarithm on the right-hand side (RHS) of (A6) as follows:

(A7) $$\begin{array}{c} \frac{P_{Z^{n}F^{n}|U^{n}}}{Q_{Z^{n}F^{n}}}\overset{\left(a\right)}{=}\frac{P_{Z^{n}F^{n}U^{n}}}{P_{U^{n}}}\frac{1}{P_{Z^{n}}Q_{F^{n}}}\overset{\left(b\right)}{=}\frac{P_{Z^{n}F^{n}U^{n}}}{P_{Z^{n}U^{n}}}\frac{1}{Q_{F^{n}}}=\frac{P_{F^{n}|Z^{n}U^{n}}}{Q_{F^{n}}}, \end{array}$$

where (a) is due to the fact that $F^{n}$ and $Z^{n}$ are independent when a meaningless discussion is transmitted, which has a pmf denoted by $Q_{F^{n}}$; (b) comes the fact that $U^{n}$ is independent of $Z^{n}$ by selection, i.e., $P_{Z^{n}U^{n}}=P_{Z^{n}}P_{U^{n}}$.

Note that even though $Z^{n}$ and $F^{n}$ are independent and $Z^{n}$ and $U^{n}$ are independent by assumption, that does not mean $Z^{n}$, $F^{n}$, and $U^{n}$ are necessarily generated according to $P_{Z^{n},\;F^{n},\;U^{n}}=P_{Z^{n}}P_{F^{n},\;U^{n}}$ or $P_{Z^{n},\;F^{n},\;U^{n}}=P_{Z^{n}}P_{F^{n}}P_{U^{n}}$. In fact, since pairwise independence does not imply mutual independence ([19] Chapter 7.1, 7.2), there exists joint distribution $P_{Z^{n},\;F^{n},\;U^{n}}$ such that we can invoke tools from the joint asymptotic equipartition property [20].

Then, we can rewrite (A6) as follows:

(A8) $$\begin{array}{c} E_{C}[D(P_{Z^{n}F^{n}|K}\left|\right|Q_{Z^{n}F^{n}}|P_{K})]\leq E_{Z^{n}F^{n}U^{n}}\left[\log\left(\frac{P_{F^{n}|Z^{n}U^{n}}}{L_{1}Q_{F^{n}}}+1\right)\right]. \end{array}$$

The RHS of (A8) can be discussed in two cases as follows similar to [3], according to whether $\left(z^{n},\;f^{n},\;u^{n}\right)$ are jointly typical or not:

$$\begin{array}{cc} d_{1} & =\sum_{\begin{array}{c} (z^{n},\;f^{n},\;u^{n})\in \\ T_{\delta }^{n}\left(P_{Z^{n},\;F^{n},\;U^{n}}\right) \end{array}}P_{Z^{n}F^{n}U^{n}}\left(z^{n},f^{n},u^{n}\right)\log\left(\frac{P_{F^{n}|U^{n}Z^{n}}\left(f^{n}|u^{n}z^{n}\right)}{L_{1}Q_{F^{n}}\left(f^{n}\right)}+1\right),\\ d_{2} & =\sum_{\begin{array}{c} (z^{n},\;f^{n},\;u^{n})\notin \\ T_{\delta }^{n}\left(P_{Z^{n},\;F^{n},\;U^{n}}\right) \end{array}}P_{Z^{n}F^{n}U^{n}}\left(z^{n},f^{n},u^{n}\right)\log\left(\frac{P_{F^{n}|U^{n}Z^{n}}\left(f^{n}|u^{n}z^{n}\right)}{L_{1}Q_{F^{n}}\left(f^{n}\right)}+1\right), \end{array}$$

where $T_{\delta }^{n}$ follows the δ-robust typicality [12] definition for the subsequent derivation.

The Chernoff bound and an important upper bound, which will be used later, are restated in the following.

**Lemma A1.** 
*(Chernoff bound ([12] Lemma 16) For every a∈X, $x^{n}\in X^{n}$, and δ>0,*

(A9) $$\begin{array}{c} P\left(\frac{N(a|x^{n})}{n}\leq \left(1+\delta \right)P_{X}\left(a\right)\right)\leq e^{-\delta^{2}P_{X}\left(a\right)n/3}. \end{array}$$

**Lemma A2.** 
*(Upper bound of the probability of a non-typical set ([12] Lemma 17)*

(A10) $$\begin{array}{c} P\left(x^{n}\notin T_{\delta }^{\left(n\right)}\right)\leq 2\left|S_{X}\right|e^{-\delta^{2}\mu_{X}n/3}, \end{array}$$

*where $S_{X}≜\left\{x\in X:\;P\left(x\right)>0\right\}$ and $\mu_{x}≜\min_{x\in S_{X}}P\left(x\right)$.*

Next, we derive the constraint (The constraint that Bob should successfully decode both the secret and confusion messages is a point-to-point transmission without secrecy, which can be seen from [12]. Therefore, we omit the proof here.) on $R_{1}$ as follows:

(A11) $$\begin{array}{cc} d_{1} & \overset{\left(a\right)}{\leq }\left(\sum_{\begin{array}{c} (z^{n},\;f^{n},\;u^{n})\in \\ T_{\delta }^{n}\left(P_{Z^{n}F^{n}U^{n}}\right) \end{array}}P_{Z^{n}F^{n}U^{n}}\left(z^{n},f^{n},u^{n}\right)\right)\log\left(1+\frac{2^{-n[H(F|UZ)-\delta ]}}{L_{1}2^{-n(1+\epsilon )H(F)}}\right)\\ \; & \overset{\left(b\right)}{\leq }\log\left(1+\frac{2^{-n[H(F|UZ)-\delta ]}}{L_{1}2^{-n(1+\epsilon )H(F)}}\right)\\ \; & \overset{\left(c\right)}{=}\log\left(1+2^{-n\left(R_{1}-I\left(F;UZ\right)-\epsilon^{\prime }\right)}\right), \end{array}$$

where (a) comes from ([12] Lemma 18, Lemma 20); (b) comes from the fact that the total probability of the jointly typical set is smaller than one; (c) is from definition of $L_{1}$ and $\epsilon^{\prime }≜\epsilon \left[1+H\left(F\right)\right]=\epsilon \left[1+H\left(U\right)\right]$. After that, we have $d_{1}\to 0$ if n→∞ when the following constraint is fulfilled:

(A12) $$\begin{array}{c} R_{1}>I\left(UZ;F\right)+\epsilon^{\prime }\overset{\left(a\right)}{=}I\left(UZ;U⊕X\right)+\epsilon^{\prime }\overset{\left(b\right)}{=}H\left(U\right)-H\left(X|Z\right)+\epsilon^{\prime }, \end{array}$$

where (a) comes from a specific use of the public discussion following ([16] Theorem 3) and ⊕ is the modulo addition in X. We can follow the argument in ([21] Appendix B) in order to apply the crypto lemma in (A12) or (A14) to unbounded *X* like the Gaussian case. (b) comes from the fact that *U* is uniformly distributed with the crypto lemma. In addition, we can derive that $d_{2}\to 0$ as n→∞ as follows:

(A13) $$\begin{array}{cc} d_{2} & \overset{\left(a\right)}{\leq }\sum_{\left(z^{n},u^{n},\;f^{n}\right)\notin T_{\delta }^{n}\left(P_{Z^{n},U^{n},F^{n}}\right)}P_{Z^{n}F^{n}U^{n}}\left(z^{n},f^{n},u^{n}\right)\log\left(\frac{1}{Q_{F^{n}}^{n}\left(f^{n}\right)}+1\right)\\ \; & \overset{\left(b\right)}{\leq }\sum_{\left(z^{n},u^{n},\;f^{n}\right)\notin T_{\delta }^{n}\left(P_{Z^{n},U^{n},F^{n}}\right)}P_{Z^{n}F^{n}U^{n}}\left(z^{n},f^{n},u^{n}\right)\log\left(\frac{1}{\mu_{f^{n}}^{n}}+1\right)\\ \; & \overset{\left(c\right)}{\leq }\sum_{\left(z^{n},u^{n},\;f^{n}\right)\notin T_{\delta }^{n}\left(P_{Z^{n},U^{n},F^{n}}\right)}P_{Z^{n}F^{n}U^{n}}\left(z^{n},f^{n},u^{n}\right)n\log\left(\frac{1}{\mu_{f}}+1\right)\\ \; & \overset{\left(d\right)}{=}nPr\left(\left(z^{n},u^{n},\;f^{n}\right)\notin T_{\delta }^{n}\left(P_{Z^{n},U^{n},F^{n}}\right)\right)\log\left(\frac{1}{\mu_{f}}+1\right)\\ \; & \overset{\left(e\right)}{\leq }2n\left|S_{ZUF}\right|e^{-\delta^{2}\mu_{ZUF}n/3}\log\left(\frac{1}{\mu_{f}}+1\right), \end{array}$$

where (a) is due to the fact that $P_{F^{n}|U^{n}Z^{n}}\left(f^{n}|u^{n}z^{n}\right)\leq 1$, and therefore, $P_{F^{n}|U^{n}Z^{n}}\left(f^{n}|u^{n}z^{n}\right)/L_{1}\leq 1$; (b) is by lower bounding $Q_{F^{n}}^{n}\left(f^{n}\right)$ with $\mu_{f}=\min_{f^{n}\in S_{F^{n}}}Q_{F^{n}}\left(f^{n}\right)$, where $S_{F^{n}}≜\left\{f^{n}\in X^{n}:P\left(f^{n}\right)>0\right\}$; (c) by simple algebra; (d) is by definition of probability; (e) is by Lemma A2. Note that $\mu_{f}$ in (A13) is a constant, but not a function of *n*. Therefore, the RHS of (A13) can be easily seen to vanish exponentially fast, if n→∞. Then, from (A11) and (A13), it is clear that (4) and (5) are fulfilled.

By constructing the CWTC, the following rate between Alice and Bob is achievable:

(A14) $$\begin{array}{cc} n(R+R_{1}) & \leq I(U^{n}⊕X^{n},Y^{n};U^{n})\\ \; & =H\left(Y^{n},U^{n}⊕X^{n}\right)-H\left(Y^{n},U^{n}⊕X^{n}|U^{n}\right)\\ \; & \overset{\left(a\right)}{=}H\left(Y^{n}\right)+H\left(U^{n}\right)-\;H\left(X^{n},Y^{n}\right)\\ \; & =H\left(U^{n}\right)\;-\;H\left(X^{n}|Y^{n}\right), \end{array}$$

where (a) again comes from the crypto lemma with the selection of $U^{n}$ being independent of $Y^{n}$. Then, from (A12) and (A14), the achievable stealthy strong SK rate can be derived as follows:

(A15) $$\begin{array}{cc} nR & \leq H\left(U^{n}\right)-H\left(X^{n}|Y^{n}\right)-nR_{1}\\ \; & \overset{\left(a\right)}{<}n\left[H\left(X|Z\right)-H\left(X|Y\right)\right]\\ \; & =n[I(X;Y)-I(X;Z)], \end{array}$$

where (a) is by plugging (A12) with the assumption of a memoryless common randomness, which is independent and identically distributed (i.i.d.). We can interchange the roles of *X* and *Y* to get I(Y;X)−I(Y;Z), which completes the proof.

**Remark** **A1.**
*In addition to the channel resolvability scheme, we can also attain the proof by a modified SWC scheme, which is sketched as follows. We can first construct a binary auxiliary random variable S, which selects the meaningful or meaningless discussion when S=1 and S=0, respectively. The stealth constraint is to avoid Willie successfully guessing the realization of S and can be formulated as $I(S;Z^{n}F^{n})\leq \epsilon .$ By the data processing inequality for divergence [17], we can have the inequality $I\left(S;Z^{n}F^{n}\right)\leq D(P_{KZ^{n}F^{n}}\left|\right|P_{K}Q_{Z^{n}}Q_{F^{n}})$, which is an effective secrecy constraint of the considered SKG model. It is the counterpart to the one of the wiretap channel with stealth constraint [3]. We can then construct two binning codebooks for S=0 and S=1, where in each case, there is one corresponding binning codebook, in order to fulfill the secrecy constraint. In the error analysis, there are two cases: (1) when S=1, the probability of Alice and Bob having different keys; (2) when S=0, the probability of Bob generating a key that is not null. By vanishing enforcing the average error probability, we can attain the result in Theorem 1.*

**Remark** **A2.**
*In the analysis by the WTC scheme in Appendix A, we derive the stealthy strong SK rate by combining a tool based one channel resolvability developed in [3] with the CWTC. In particular, we first derive an upper bound of the averaged stealth secrecy constraint (A5) by the random coding analysis. By enforcing the upper bound to vanish, we can derive the constraints on the stealthy strong SK rate and the confusion rate in the codebook design. By this scheme, we can proceed with the derivation based on the result of the wiretap channel.*

## Appendix B. Proof of (A6)

In the following, we show the tedious derivation for (A6) from channel resolvability [3]:

$$\begin{array}{cc} \; & E_{C}[D(P_{Z^{n}F^{n}|K}\left|\right|Q_{Z^{n}F^{n}}|P_{K})]\\ \; & \overset{\left(a\right)}{=}E_{C}[D(P_{Z^{n}F^{n}|M}\left|\right|Q_{Z^{n}F^{n}}|P_{M})]\\ \; & \overset{\left(b\right)}{=}E_{C,M}\left[\sum_{z^{n},F^{n}}P_{Z^{n},F^{n}|M}\left(z^{n},f^{n}|M=m\right)\log\left(\frac{P_{Z^{n},F^{n}|M}\left(z^{n},f^{n}|M=m\right)}{Q_{Z^{n},F^{n}}\left(z^{n},f^{n}\right)}\right)\right]\\ \; & \overset{\left(c\right)}{=}E_{C,M}\left[\sum_{z^{n},F^{n}}\sum_{w=1}^{L_{1}}\frac{1}{L_{1}}P_{Z^{n},F^{n}|U^{n}}\left(z^{n},f^{n}|u^{n}\left(m,w\right)\right)\log\left(\frac{\sum_{l=1}^{L_{1}}\frac{1}{L_{1}}P_{Z^{n},F^{n}|U^{n}}\left(z^{n},f^{n}|u^{n}\left(m,l\right)\right)}{Q_{Z^{n},F^{n}}\left(z^{n},f^{n}\right)}\right)\right]\\ \; & \overset{\left(d\right)}{=}\frac{1}{LL_{1}}E_{C}\left[\sum_{z^{n},F^{n}}\sum_{m=1}^{L}\sum_{w=1}^{L_{1}}P_{Z^{n},F^{n}|U^{n}}\left(z^{n},f^{n}|u^{n}\left(m,w\right)\right)\log\left(\frac{\sum_{l=1}^{L_{1}}Q_{Z^{n},F^{n}|U^{n}}\left(z^{n},f^{n}|u^{n}\left(m,l\right)\right)}{L_{1}P_{Z^{n},F^{n}}\left(z^{n},f^{n}\right)}\right)\right]\\ \; & \overset{\left(e\right)}{=}\frac{1}{LL_{1}}E_{C}\left[\sum_{z^{n},F^{n}}\sum_{m=1}^{L}\sum_{w=1}^{L_{1}}P_{Z^{n},F^{n}|U^{n}}\left(z^{n},f^{n}|u^{n}\left(m,w\right)\right)\log\left(\frac{A_{m}\left(z^{n},f^{n}|u^{n}\right)}{B(z^{n},f^{n})}\right)\right]\\ \; & \overset{\left(f\right)}{=}\frac{1}{LL_{1}}\sum_{u^{n}\left(1,1\right)}\cdots \sum_{u^{n}\left(L,L_{1}\right)}\prod_{m=1,w=1}^{L,L_{1}}P_{U}^{n}\left(u^{n}\left(m,w\right)\right)\left[\sum_{z^{n},F^{n}}\sum_{m=1}^{L}\sum_{w=1}^{L_{1}}P_{Z^{n},F^{n}|U^{n}}\left(z^{n},f^{n}|u^{n}\right)\log\left(\frac{A_{m}\left(z^{n},f^{n}|u^{n}\right)}{B(z^{n},f^{n})}\right)\right]\\ \; & \overset{\left(g\right)}{=}\frac{1}{LL_{1}}\sum_{u^{n}\left(1,1\right)}\cdots \sum_{u^{n}\left(L,L_{1}\right)}\prod_{m=1,w=1}^{L,L_{1}}P_{U}^{n}\left(u^{n}\left(m,w\right)\right)\\ \; & \sum_{z^{n},F^{n}}\left[\begin{array}{ccc} (P_{Z^{n},F^{n}|U^{n}}\left(\cdot |u^{n}\left(1,1\right)\right)+ & \cdots & +P_{Z^{n},F^{n}|U^{n}}\left(\cdot |u^{n}\left(1,L_{1}\right)\right))\cdot \log\left(\frac{A_{1}\left(z^{n},f^{n}|u^{n}\right)}{B(z^{n},f^{n})}\right)+\\ \vdots & \ddots & \vdots \\ (P_{Z^{n},F^{n}|U^{n}}\left(\cdot |u^{n}\left(L,1\right)\right) & \cdots & +P_{Z^{n},F^{n}|U^{n}}\left(\cdot |u^{n}\left(L,L_{1}\right)\right))\cdot \log\left(\frac{A_{L}\left(z^{n},f^{n}|u^{n}\right)}{B(z^{n},f^{n})}\right) \end{array}\right]\\ \; & \overset{\left(h\right)}{=}\frac{1}{LL_{1}}\sum_{u^{n}\left(1,1\right)}\cdots \sum_{u^{n}\left(L,L_{1}\right)}\\ \; & \sum_{z^{n},F^{n}}\left[\begin{array}{c} \left(P_{Z^{n},F^{n},U^{n}}\left(\cdot ,u^{n}\left(1,1\right)\right)\prod_{m\neq 1,w\neq 1}^{L,L_{1}}P_{U}^{n}\left(u^{n}\left(m,w\right)\right)+\cdots +P_{Z^{n},F^{n},U^{n}}\left(\cdot ,u^{n}\left(1,L_{1}\right)\right)\prod_{m\neq 1,w\neq 1}^{L,L_{1}}P_{U}^{n}\left(u^{n}\left(m,w\right)\right)\right)\cdot \\ \log\left(\frac{A_{1}\left(z^{n},f^{n}|u^{n}\right)}{B(z^{n},f^{n})}\right)+\cdots \\ \left(P_{Z^{n},F^{n},U^{n}}\left(\cdot ,u^{n}\left(L,1\right)\right)\prod_{m\neq 1,w\neq 1}^{L,L_{1}}P_{U}^{n}\left(u^{n}\left(m,w\right)\right)+\cdots +P_{Z^{n},F^{n},U^{n}}\left(\cdot ,u^{n}\left(L,L_{1}\right)\right)\prod_{m\neq 1,w\neq 1}^{L,L_{1}}P_{U}^{n}\left(u^{n}\left(m,w\right)\right)\right)\cdot \\ \log\left(\frac{A_{L}\left(z^{n},f^{n}|u^{n}\right)}{B(z^{n},f^{n})}\right) \end{array}\right] \end{array}$$

(A16) $$\begin{array}{cc} \; & \overset{\left(i\right)}{=}\frac{1}{LL_{1}}\sum_{z^{n},F^{n}}\left[\begin{array}{c} \sum_{u^{n}\left(1,1\right)}P_{Z^{n},F^{n},U^{n}}\left(\cdot ,u^{n}\left(1,1\right)\right)E_{{\underline{U}}^{n}\setminus {\underline{U}}^{n}\left(1,1\right)}\left[\log\left(\frac{A_{1}\left(z^{n},f^{n}|u^{n}\right)}{B(z^{n},f^{n})}\right)\right]+\cdots +\\ \sum_{u^{n}\left(k,l\right)}P_{Z^{n},F^{n},U^{n}}\left(\cdot ,u^{n}\left(k,l\right)\right)E_{{\underline{U}}^{n}\setminus {\underline{U}}^{n}\left(k,l\right)}\left[\log\left(\frac{{}_{k}\left(z^{n},f^{n}|u^{n}\right)}{B(z^{n},f^{n})}\right)\right]+\cdots +\\ \sum_{u^{n}\left(L,L_{1}\right)}P_{Z^{n},F^{n},U^{n}}\left(\cdot ,u^{n}\left(L,L_{1}\right)\right)E_{{\underline{U}}^{n}\setminus {\underline{U}}^{n}\left(L,L_{1}\right)}\left[\log\left(\frac{A_{L}\left(z^{n},f^{n}|u^{n}\right)}{B(z^{n},f^{n})}\right)\right] \end{array}\right]\\ \; & \overset{\left(j\right)}{=}\frac{1}{LL_{1}}\sum_{z^{n},F^{n}}\underset{\left(a,b)=(1,1\right)}{\sum^{\left(L,L_{1}\right)}}\sum_{u^{n}\left(a,b\right)}P_{Z^{n},F^{n},U^{n}}\left(\cdot ,u^{n}\left(a,b\right)\right)E_{{\underline{U}}^{n}\setminus {\underline{U}}^{n}\left(a,b\right)}\left[\log\left(\frac{A_{a}\left(z^{n},f^{n}|u^{n}\right)}{B(z^{n},f^{n})}\right)\right]\\ \; & \overset{\left(k\right)}{\leq }\frac{1}{LL_{1}}\sum_{z^{n},F^{n}}\underset{\left(a,b)=(1,1\right)}{\sum^{\left(L,L_{1}\right)}}\sum_{u^{n}\left(a,b\right)}P_{Z^{n},F^{n},U^{n}}\left(\cdot ,u^{n}\left(a,b\right)\right)\log\left(E_{{\underline{U}}^{n}\setminus {\underline{U}}^{n}\left(a,b\right)}\left[\left(\frac{A_{a}\left(z^{n},f^{n}|u^{n}\right)}{B(z^{n},f^{n})}\right)\right]\right)\\ \; & \overset{\left(l\right)}{=}\frac{1}{LL_{1}}\sum_{z^{n},F^{n}}\underset{\left(a,b)=(1,1\right)}{\sum^{\left(L,L_{1}\right)}}\sum_{u^{n}\left(a,b\right)}P_{Z^{n},F^{n},U^{n}}\left(\cdot ,u^{n}\left(a,b\right)\right)\log\left(\frac{P_{Z^{n},F^{n}|U^{n}}\left(\cdot |u^{n}\left(a,b\right)\right)+\sum_{s\neq b}\sum_{u^{n}}^{\left(a,s\right)}P_{Z^{n},F^{n},U^{n}}\left(\cdot ,u^{n}\left(a,s\right)\right)}{B(z^{n},f^{n})}\right)\\ \; & \overset{\left(m\right)}{\leq }\frac{1}{LL_{1}}\sum_{z^{n},F^{n}}\underset{\left(a,b)=(1,1\right)}{\sum^{\left(L,L_{1}\right)}}\sum_{u^{n}\left(a,b\right)}P_{Z^{n},F^{n},U^{n}}\left(\cdot ,u^{n}\left(a,b\right)\right)\log\left(\frac{P_{Z^{n},F^{n}|U^{n}}\left(\cdot |u^{n}\left(a,b\right)\right)+\sum_{\left(L,L_{1}\right)}^{\left(r,s)=(1,1\right)}\sum_{u^{n}}^{\left(r,s\right)}P_{Z^{n},F^{n},U^{n}}\left(\cdot ,u^{n}\left(r,s\right)\right)}{B(z^{n},f^{n})}\right)\\ \; & \overset{\left(n\right)}{\leq }\frac{1}{LL_{1}}\sum_{z^{n},F^{n}}\underset{\left(a,b)=(1,1\right)}{\sum^{\left(L,L_{1}\right)}}\sum_{u^{n}\left(a,b\right)}P_{Z^{n},F^{n},U^{n}}\left(\cdot ,u^{n}\left(a,b\right)\right)\log\left(\frac{P_{Z^{n},F^{n}|U^{n}}\left(\cdot |u^{n}\left(a,b\right)\right)}{B(z^{n},f^{n})}+1\right)\\ \; & \overset{\left(o\right)}{=}E_{Z^{n}F^{n}U^{n}}\left[\log\left(\frac{P_{Z^{n}F^{n}|U^{n}}}{L_{1}Q_{Z^{n}F^{n}}}+1\right)\right], \end{array}$$

where (a) is by constructing a CWTC, such that the key *K* is interchangeable with the message *M*; (b) is by definition of the conditional K-L distance ([17] Definition 2.2); (c) is due to the fact that $P_{Z^{n},F|M}$ is the marginalization of $P_{Z^{n},F^{n}|U^{n}}$ with respect to *w*, which is the index of the confusion message; in (d), we expand the expectation with respect to *M*; (e) is by defining $\sum_{l=1}^{L_{1}}P_{Z^{n},F^{n}|U^{n}}\left(z^{n},f^{n}|u^{n}\left(m,l\right)\right)$ and $L_{1}Q_{Z^{n},F^{n}}\left(z^{n},f^{n}\right)$ by $A_{m}\left(z^{n},f^{n}|u^{n}\right)$ and $B(z^{n},f^{n})$, respectively, to simplify the expression, where m=1,⋯,L; (f) is by definition of the expectation over ${\left\{U^{n}\left(m,w\right)\right\}}_{m=1,w=1}^{L,L_{1}}$. Since $\left\{U^{n}\left(m,w\right)\right\}$ are generated independently and identically according to $P_{U}^{n}$, the joint distribution of codewords in a codebook is the product of marginal distributions; in (g), we expand the summation with respect to *m* and *w*; in (h), we expand the product according to the form in step (g); in (i), we collect terms to form the expectation $E_{{\underline{U}}^{n}\setminus {\underline{U}}^{n}\left(k,l\right)}$; in (j), we collect the terms by introducing additional indices (a,b); in (k), we apply Jensen’s inequality to the logarithm; (l) is by expanding the expectation $E_{{\underline{U}}^{n}\setminus {\underline{U}}^{n}\left(k,l\right)}$; (m) is by adding the term $P_{Z^{n}F^{n}U^{n}}\left(z^{n},f^{n},u^{n}\left(a,b\right)\right)$; (n) is by the definition of marginalization over $P_{Z^{n}F^{n}U^{n}}$ with respect to $U^{n}$. In particular, the second term on the RHS of the numerator in (m) becomes $E_{U^{n}}\left[P_{Z^{n},F^{n}|U^{n}}\right]=Q_{Z^{n}F^{n}}$ from (A3); (o) is by definition of the expectation.

## Appendix C. Proof of Theorem 2

We sketch the proof as follows in three steps, while the main idea is summarized in Figure A1. The key is to show that the stochastically degraded source $\left(X,\tilde{Y},\tilde{Z}\right)$ implies that the corresponding CWTC [16] is also stochastically degraded, then the secret key capacity $C_{SK}$ of the source is the same as the secrecy capacity of a CWTC constructed from a physically degraded source (X,Y,Z). The first step is to construct the CWTC of the source (X,Y,Z) and prove that, if X−Y−Z, then $U-Y^{\prime }-Z^{\prime }$, i.e., the corresponding CWTC is also physically degraded, where *U* is the conceptual code symbol, uniformly distributed in X and independent of (X,Y,Z). The equivalently received signals at Bob and Willie in the CWTC are $Y^{\prime }≜\left(Y,U⊕X\right)$ and $Z^{\prime }≜\left(Z,U⊕X\right)$, respectively, while U⊕X is the signal transmitted through the public channel. The second step is to construct a stochastically degraded source $\left(X,\tilde{Y},\tilde{Z}\right)$ from (X,Y,Z). After that, we construct the corresponding CWTC of $\left(X,\tilde{Y},\tilde{Z}\right)$ as $\left(U,Y^{\prime \prime },Z^{\prime \prime }\right)$, where $Y^{\prime \prime }≜\left(\tilde{Y},U⊕X\right)$ and $Z^{\prime \prime }≜\left(\tilde{Z},U⊕X\right)$. The third step is to show that the CWTC described by $\left(U,Y^{\prime \prime },Z^{\prime \prime }\right)$ has the same marginals as the CWTC described by $\left(U,Y^{\prime },Z^{\prime }\right)$, i.e., the two CWTC’s have the same secrecy capacity. In addition to the fact that stochastic degradedness is no stronger than the physical degradedness, this results in that the former one should not have a higher secret key capacity than the latter one. We then know that the sources (X,Y,Z) and $\left(X,\tilde{Y},\tilde{Z}\right)$ have the same secret key capacity, which completes the proof.

In the following, we will prove that the stochastically degraded random source $\left(X,\tilde{Y},\tilde{Z}\right)$ implies that the corresponding CWTC [16] is also stochastically degraded, which is constructed by the corresponding physically degraded source (X,Y,Z). We start from constructing the CWTC of the random source (X,Y,Z), where the equivalently received signals at Bob and Willie are $Y^{\prime }≜\left(Y,U⊕X\right)$ and $Z^{\prime }≜\left(Z,U⊕X\right)$, respectively. If X−Y−Z, then $U-Y^{\prime }-Z^{\prime }$, i.e., the CWTC is also a physically degraded one, which can be shown as follows:

(A17) $$\begin{array}{cc} I\left(U;Z^{\prime }|Y^{\prime }\right)\overset{\left(a\right)}{=} & H(Z,U⊕X|Y,U⊕X)-H(Z,U⊕X|Y,U⊕X,U)\\ \overset{\left(b\right)}{=} & H(Z|Y)-H(Z|Y,U⊕X,U)\\ \overset{\left(c\right)}{=} & H(Z|Y)-H(Z|Y,X,U)\\ \overset{\left(d\right)}{=} & H(Z|Y)-H(Z|Y,X)\\ \overset{\left(e\right)}{=} & 0, \end{array}$$

where (a) is by the definition of $Y^{\prime }$ and $Z^{\prime }$; (b) is by the crypto lemma [22], and *U* is selected to be independent of *Y* and *Z*; (c) is from the fact that given *U*, we can know *X* from U⊕X; (d) is by the same reason as (b); (e) is due to X−Y−Z and by the definition of conditional mutual information.

Now, we consider the stochastically degraded source of common randomness $\left(X,\tilde{Y},\tilde{Z}\right)$ fulfilling $P_{\tilde{Y}|X}=P_{Y|X}$ and $P_{\tilde{Z}|X}=P_{Z|X}$. Similar to the first step, we construct the CWTC from $\left(X,\tilde{Y},\tilde{Z}\right)$, namely $\{\left(U,Y^{\prime \prime },Z^{\prime \prime }\right),$$\;P_{U,Y^{\prime \prime },Z^{\prime \prime }}=P_{U}P_{Y^{\prime \prime },Z^{\prime \prime }|U}\}$, where $Y^{\prime \prime }≜\left(\tilde{Y},U⊕X\right)$ and $Z^{\prime \prime }≜\left(\tilde{Z},U⊕X\right)$ are the equivalent channel outputs at Bob and Willie, respectively. To prove that the two CWTC’s $P_{Y^{\prime },Z^{\prime }|U}$ and $P_{Y^{\prime \prime },Z^{\prime \prime }|U}$ are equivalent, we can invoke the same marginal property in ([11] Theorem 16.6) to prove that $P_{Y^{\prime \prime }|U}=\;P_{Y^{\prime }|U}$ and $P_{Z^{\prime \prime }|U}=P_{Z^{\prime }|U}$, which is shown in the following. By the definitions of $Y^{\prime \prime }$ and $Y^{\prime }$, we know that $P_{Y^{\prime \prime }|U=u}=P_{\tilde{Y},u⊕X}≜P_{\tilde{Y},X_{u}}$ and $P_{Y^{\prime }|U=u}=P_{Y,u⊕X}≜P_{Y,X_{u}}$, respectively, where we define $X_{u}$ as u⊕X. Note that due to the closed operation ⊕ in X, the distribution of $X_{u}$ is a left circular shift of that of *X* by *u*. Instead of directly proving $P_{\tilde{Y},X_{u}}=P_{Y,X_{u}}$, we can equivalently prove $P_{\tilde{Y}|X_{u}}=P_{Y|X_{u}},\;\forall \;u$, as follows:

(A18) $$P_{\tilde{Y}|X_{u}=x}=P_{\tilde{Y}|X=x^{\prime }}=P_{Y|X=x^{\prime }}=P_{Y|X_{u}=x},$$

where the second equality is due to the assumption of $\left(X,\tilde{Y},\tilde{Z}\right)$ forming a stochastically degraded source from the physically degraded one (X,Y,Z). Therefore, $P_{Y^{\prime \prime }|U}=P_{Y^{\prime }|U}$. Similarly, we can derive $P_{Z^{\prime \prime }|U}=P_{Z^{\prime }|U}$. Hence, we prove that the CWTC of a stochastic degraded source is also a degraded CWTC and the two CWTC’s have the same secrecy capacity.

Note that due to X−Y−Z, we can derive the key capacity $C_{SK}$ from the corresponding CWTC, not just an achievable key rate. For the source $\left(X,\tilde{Y},\tilde{Z}\right)$, till now, we may only claim the achievable secret key rate, but not the secret key capacity, namely $C_{SK}^{\prime }$, is the same as $C_{SK}$. That is because the CWTC is in general only an achievable scheme to derive the secret key rate. However, due to the fact that the stochastic degradedness is more general than the physical degradedness, i.e., less stringent on characterizing the order between $X,\;\tilde{Y}$ and $\tilde{Z}$, the stochastic degradedness cannot result in a larger secret key capacity than the physically degraded one. Therefore, we attain that $C_{SK}^{\prime }=C_{SK}=I\left(X;Y\right)-I\left(X;Z\right)$, which completes the proof.
